# MAIC–10 brief quality checklist for publications using artificial intelligence and medical images

**DOI:** 10.1186/s13244-022-01355-9

**Published:** 2023-01-16

**Authors:** Leonor Cerdá-Alberich, Jimena Solana, Pedro Mallol, Gloria Ribas, Miguel García-Junco, Angel Alberich-Bayarri, Luis Marti-Bonmati

**Affiliations:** 1 Clinical Medical Imaging Area and Biomedical Imaging Research Group (GIBI230-PREBI), Hospital Universitario y Politécnico La Fe – Instituto de Investigación Sanitaria La Fe, Valencia, Spain; 2 Quantitative Imaging Biomarkers in Medicine, Quibim SL, Valencia, Spain

**Keywords:** Artificial intelligence, Medical imaging, Checklist, Software as a medical device

## Abstract

The use of artificial intelligence (AI) with medical images to solve clinical problems is becoming increasingly common, and the development of new AI solutions is leading to more studies and publications using this computational technology. As a novel research area, the use of common standards to aid AI developers and reviewers as quality control criteria will improve the peer review process. Although some guidelines do exist, their heterogeneity and extension advocate that more explicit and simple schemes should be applied on the publication practice. Based on a review of existing AI guidelines, a proposal which collects, unifies, and simplifies the most relevant criteria was developed. The MAIC-10 (Must AI Criteria-10) checklist with 10 items was implemented as a guide to design studies and evaluate publications related to AI in the field of medical imaging. Articles published in *Insights into Imaging* in 2021 were selected to calculate their corresponding MAIC-10 quality score. The mean score was found to be 5.6 ± 1.6, with critical items present in most articles, such as “Clinical need”, “Data annotation”, “Robustness”, and “Transparency” present in more than 80% of papers, while improvements in other areas were identified. MAIC-10 was also observed to achieve the highest intra-observer reproducibility when compared to other existing checklists, with an overall reduction in terms of checklist length and complexity. In summary, MAIC-10 represents a short and simple quality assessment tool which is objective, robust and widely applicable to AI studies in medical imaging.

**Supplementary Information:**

The online version contains supplementary material available at 10.1186/s13244-022-01355-9.

## Key points

- AI solutions have become an essential clinical tool in medical imaging.
- Standardised criteria are necessary to ensure quality of AI studies.
- Established criteria are often incomplete, too exhaustive, or not broadly applicable.
- A concise and reproducible quantitative checklist will help to ensure a minimum of acceptance.

**Supplementary Information:**

The online version contains supplementary material available at 10.1186/s13244-022-01355-9.

## Introduction

Artificial intelligence (AI) offers solutions that are revolutionising medicine, progressing towards the paradigm shift represented by data-driven decision making. In the field of medical imaging, an increasing number of studies are appearing in which AI tools are making important contributions towards more accurate diagnoses or more reliable prognostic estimates [1, 2]. Although there are very high expectations, the validity of many of these publications can be brought into question as the methodology used in their design is criticised [1]. Before AI-based solutions are accepted in clinical practice, appropriate methodological quality control mechanisms must be agreed upon. If this approach is implemented with a guarantee of robustness and clinical validity, AI-based solutions could be certified as medical devices (software as a medical device—SaMD) and appropriate periodic surveillance mechanisms put in place.

For decades, our group has focused on the standardisation and validation of imaging biomarkers. In recent years, AI-based implementations using convolutional neural networks (CNN) have been developed in the framework of large research projects funded by the European Commission’s Horizon 2020 programme [3, 4]. However, the poor explainability (black box effect), the small size and heterogeneity of the training data set, and the limited independent validation of the results, have generated growing concerns and a call for greater transparency and rigour to ensure more reliable AI models. Hence, there is a growing interest to draw up checklists used to verify the proper implementation of AI models in medical imaging to ensure that adequate reproducibility and clinical impact is provided prior to their use. Since different sets of criteria have been proposed, it is desirable to concisely define the minimum core standards that must be adhered to in these studies and publications.

Our aim is to formulate a guideline unifying, combining, and summarising existing ones, focusing on medical imaging and AI. Then, these quality criteria will be checked in published AI studies, assessing similarities and differences when compared to the previous existing checklists. Based on this analysis, a basic scientific quality control checklist is proposed to guarantee a minimum standard of acceptability when carrying out and evaluating AI research publications.

## Methodology

After reviewing in PubMed the 2017–2021 publications on AI methodology and quality control in Q1 high impact journals by searching the terms “Checklist”, “AI,” and “imaging”, six manuscripts proposing the use of AI checklists in clinical pathways were identified:

- Checklist for artificial intelligence in medical imaging (CLAIM), sponsored by the Radiology: Artificial Intelligence journal (Radiological Society of North America) [5, 6].
- Minimum information about clinical artificial intelligence modelling (MI-CLAIM), catalogued in the EQUATOR Network library of reporting guidelines [7].
- Radiomics quality score (RQS), developed at Maastricht University Medical Centre and widely used [8].
- Consolidated standards of reporting trials-AI (CONSORT-AI), developed by the CONSORT-AI and SPIRIT-AI Working Group and catalogued in the EQUATOR Network library [9].
- Standard protocol items recommendations for interventional trials (SPIRIT-AI), developed by the CONSORT-AI and SPIRIT-AI Working Group and catalogued in the EQUATOR Network library [10].
- Clinical AI research (CAIR) checklist, published recently by a group of researchers led by the Karolinska Institute in Sweden [11].

These manuscripts were evaluated to compare, define, and establish a simplified reference criterion for the use of AI in the medical imaging environment. These initiatives were conceived with slightly different goals and, thus, the most relevant aspects of the AI approaches that should be considered when designing studies also differ based on each perspective. The stepwise selection of the main checklist items was as follows. First, a shortlist was created including items which appeared in at least two of the checklists. Each item in the shortlist was considered by all seven authors, who voted for or against its inclusion according to the following principles: (a) low relevance for AI-focused applications (i.e. items related to general scientific methodology aspects), (b) scope limited to clinical trial methods (including aspects such as protocol registration, adverse event handling, etc.), (c) relevance in medical imaging-focused studies, (d) relationship to study reproducibility (focusing on statistical and methodological aspects), (e) expected final clinical impact, and (f) broad applicability across study types (retrospective vs prospective, hand-crafted radiomics vs deep learning, regression vs segmentation, image-only vs multi-modal data, etc.). A minimum of four authors agreement was used as a threshold for selection, without considering years of experience (mean of 11 years, 3–30 range) neither professional background (two radiologists, three data scientists, two computational scientists). Finally, the resulting set of criteria was unanimously accepted by the authors multi-disciplinary team. The final criteria were reviewed, eliminating repetitions by merging overlapping items, and simplifying definitions. The final checklist was named MAIC-10 (Must AI Criteria-10), as it represents the 10 essential criteria considered to be necessary in any AI publication with medical images. To optimise the checklist’s usability for both authors and reviewers, and with the aim to perform reliable comparisons between different checklists, MAIC-10 was also formatted to allow the easy calculation of the quality score as a percentage.

At the writing this article, at least four more checklists are in development. TRIPOD-AI and PROBAST-AI are two “-AI” extensions of current TRIPOD standards and PROBAST tool which are focused on the reporting of multivariable prediction models for diagnosis or prognosis and as a tool for assessment in risk of bias on prediction models, respectively [12]. STARD-AI and DECIDE-AI are also -AI extensions of current standards focused on study design and in the evaluation and reporting of human factors in clinical AI studies, respectively [13]. They are more general AI checklist that will have to be evaluated after publication.

To ensure applicability when evaluating already published papers on AI and medical imaging, a representative set of articles from the journal Insights into Imaging was considered as a pilot validation and evaluation. The Q1 journal Insights into Imaging was searched for recent original articles using AI methodology and medical images using the journal’s “Search by keyword” tool. Searches were performed on the 2021 issue using the keywords “AI”, “Artificial Intelligence”, “Deep Learning”, “DL”, “Machine Learning”, “ML”, “CNN”, “Neural Network”, and “Predictive models”. After excluding reviews and opinion letters, 33 original articles were identified. This list was further filtered and narrowed down to 10 relevant original articles dealing with AI and medical images developments [14–23]. The selected papers were critically assessed for compliance with each item included in the MAIC-10 checklist. Finally, MAIC-10 was compared with the six initial checklists based on their applicability, usability and objectivity, and overall scores were obtained by normalising each score as a percentage. These 10 manuscripts were scored twice by 3 of the authors (J.S., P.M., G.R.) in agreement, with 4 months between each evaluation. Intra-observer reproducibility was then calculated in terms of the repeatability coefficient (${\text{RC}} = 1.96 \cdot \sqrt {2 \cdot \sigma_{{{\text{intra}}}}^{2} }$), where σintra is the intra-observer standard deviation. In cases with only two measurements, this expression can be approximated as the average (mean) sample standard deviation of multiple sets of repeated measurements (${\text{RC}} = 1.96\cdot{ }\sqrt {\frac{{\sum \left( {m_{2} - m_{1} } \right)^{2} }}{n}}$), where m1 and m2 are the two scores obtained on each publication by the same observer, and n is the number of total publications (*n* = 10). The reason behind this simplification is because the variance of two observations is equal to half the square of their difference. In general terms, the smaller the repeatability coefficient is, the better, as it corresponds to the expected difference to be obtained in 95% of cases when performing the same measurement twice under the same conditions. A lineal regression was applied to estimate the correlation between the quality scores obtained with MAIC-10 and with the CLAIM, MI-CLAIM, and CAIR checklists, respectively, and the corresponding *R*^2^ scores were reported. All analyses were performed with Microsoft Excel [24].

## Results

Overall, 10 criteria were finally proposed after reviewing the selected guidelines [5, 7–11] and summarised into a concise MAIC-10 checklist (Table 1), which allows the straightforward calculation of a quality score. These manuscripts quality criteria are described in more detail below.

1. Clinical need. Clearly defines the target clinical problem to be addressed and justifies the use and utility of an AI approach to solve this problem, both from a technical perspective and in terms of the added value to the patient. Defines also previous statistics and AI applications to the problem.
2. Study Design. Indicates the type of study carried out and the number of involved centres. In an AI setting, most studies will be observational on data collected without any intervention. Describes how the target study population was identified and the main characteristics of the patients included in the study. Specifies the recruitment period, the inclusion and exclusion criteria, as well as the type and number of expected imaging studies and equity issues. Additionally, provides a sample size estimate and describes how it was calculated. Establishes the level of confidence to operate with and the approximate number of predictors expected to explain the clinical data and to propose models that might enable the results to be extrapolated to the general population.
3. Safety and Privacy. Defines the ethical, legal, and social implications (ELSI) of the study. Explicitly, indicates if the study was approved by an ethics committee and whether informed consent was obtained. It also describes the process used for de-identification (pseudonymisation or anonymisation) of the data, as well as any cybersecurity aspects and privacy issues that apply.
4. Data Curation. Defines the origin of the data used in the study and the procedures used for its extraction from medical records, PACS (picture archiving and communication systems) or repositories, as well as any established data quality controls. Defines the use of standardised structure models (such as the common data model for electronic case report form, CDM-eCRF). Describes all the procedures implemented for organising, describing, integrating, cleaning, enhancing, transforming, and preserving data. Regarding images, describes the pre-processing applied to harmonise images and to improve image quality. The software used and the hyperparameters of each of the transformations applied should be specified.
5. Data annotation. Defines all the benchmarks used, both for the predictor variables, and for the variables that the AI aims to identify. Defines the criteria by which these reference patterns were considered as "true". Reference standards must be accepted by the scientific community when defining clinical outcomes, such as pathology results or time to survival. Describes the data annotation process, stating the methods and metrics used to assess inter- and intra-observer variability. Additionally, mentions how identified discrepancies were resolved.
6. Data Partitioning. Specifies how the data was split into training, tuning, test and validation sets, the proportion of cases that were assigned to each partition, and the reasoning behind the establishment of the partitions. Describes group characteristics and real-world data representativity. A flow chart summarising number of cases and data sets is appreciated. Describes the performance of the final AI model using a validation data set, i.e. unseen data after model training and tuning. Ideally an external independent validation data set should have been used, defined as data from centres, scanners, and protocols other than those used to train and test the model.
7. AI Model. Describes the AI model architecture and selected hyperparameters, together with the technologies, software and hardware used. The variables to be predicted (outputs) were also be defined, which may be intermediate, such as organ segmentation, or a final clinical event related to the clinical question. Indicates the metrics used to evaluate the goodness of fit of the trained model on the test data set, such as precision, accuracy or specificity [25]. Describes all the training, hyperparameter tuning and testing procedures of the AI solution in sufficient detail so that it can be replicated by other researchers in other environments. Specifies the performance criteria used to select the final model.
8. Robustness. Ensures the highest possible strength of the AI solution, defined as the quality that allows consistent and reproducible results to be obtained in clinical settings regardless of the data source. If the developed solution is not robust, it was discussed as a limitation. Discusses the main shortcomings of the model, those that become evident during the most extensive possible evaluation of its use. Describes the possible role of AI in the field of study, as well as the situations that might limit its application in clinical practice and real-world conditions.
9. Explainability. Provides methods for analysing or complementing AI models to make the internal logic and output of algorithms interpretable, making these processes understandable and meaningful. Black box effects were pointed out as limitations. AI solutions were accompanied with some form of uncertainty or confidence metric. Provides information to understand outliers or incorrect results. Explainable AI is a prerequisite to clinical deployment of AI models [26].
10. Transparency. Defines whether there is open access to the code and data used to construct and validate the AI model, allowing the results and performance claims to be verified. Open access may also be useful for transfer learning. Mentions the sources of financing, as well as any conflicts of interest held by the authors. Includes the role of sponsors, as well as authors’ autonomy.

**Table 1:** MAIC-10 checklist to assess quality of AI-based medical imaging research studies

Checklist item	Article section	Description	Reported
1. Clinical need	Introduction	The study is clearly put into context by describing the target clinical problem and any previous approaches in the literature	□
2. Study design	Materials and methods	The type of study (observational/interventional, single/multicentre) and inclusion/exclusion criteria are explicitly described, and a sample size estimate is given	□
3. Safety and privacy	Materials and methods	ELSI (ethical, legal, social implications), specifically including ethics committee approval and data de-identification issues, are discussed	□
4. Data curation	Materials and methods	Data extraction, cleaning, and transformation methods, including image pre-processing steps, are clearly described	□
5. Data annotation	Materials and methods	The ground truth reference is defined and the annotation process, including measures of inter/intra-observer variability, is described	□
6. Data partitioning	Materials and methods	Methods and criteria for data set splitting into train-tune-test-validation sets are indicated	□
7. AI model	Materials and methods, results	The AI model building methodology is sufficiently detailed by including used technologies (software and hardware), training–tuning–testing methods, performance metrics, and resulting AI model architecture	□
8. Robustness	Results, discussion	The generalizability of the AI model in real-world conditions is explicitly discussed	□
9. Explainability	Discussion	The interpretability of the model (including the use of uncertainty or confidence metrics) is explicitly discussed	□
10.Transparency	Discussion	Any possibility of access to original data sets and source code is clearly stated. Financing and conflicts of interest are detailed	□
			Score:

All the listed sub-items under the descriptions should be addressed to consider that the corresponding item has been fulfilled. The MAIC-10 checklist is structured based on the different sections of a research publication: Introduction, Materials and Methods, Results and Discussion

When assessing the quality of a study by using the MAIC-10 checklist, a score of 1 is assigned for each item with which it complies. The maximum possible score is 10. From the selected set of articles from *Insights into Imaging* published in 2021, the corresponding quality scores were calculated. A summary of this review can be found in Additional file 1. The average quality score was 5.6 ± 1.6, with most checklist items being discussed in at least half of the articles, while only one (“Study design”) was not defined in any of the studies.

The following checklist items were present in almost all papers: “Clinical need” (90% of publications), “Data annotation” (80%), “Robustness” (80%), and “Transparency” (80%). Items on clinical needs and transparency correspond to traditionally discussed aspects in scientific publications and are consistently considered during the peer review process. Therefore, high scores were expected. The high compliance rate for items on data annotation and robustness is an indicator of good-quality methodology in these AI studies. Data annotation workflows are consistently well reported and, while not all models created in the literature are highly robust, this limitation is explicitly acknowledged and discussed.

The min lacking aspects in the reviewed studies, according to MAIC-10, were the reporting of a sample size estimate (included in “Study design”), the description of data de-identification protocols (“Safety and privacy”), and multiple issues related to technical AI methodology, namely the reasoning behind the establishment of train-test-validation data set partitions, the mention of hardware used for model training, and any discussion regarding the AI model’s interpretability (covered by “Data partitioning”, “AI model”, and “Explainability”, respectively). In this regard, it must be emphasised that the calculation of sample size estimates, the choice of rationale for data set splitting, and the addition of uncertainty metrics as model outputs represent critical points which should be addressed to ensure good-quality AI studies. These items relate to statistical aspects are crucial to prove that a model is well designed and reliable. Regarding “Safety and privacy”, some of the articles mention that the medical images used for AI model training are de-identified but did not provide much detail about the anonymisation process or criteria, while data privacy issues were generally not addressed.

**Fig. 1:**
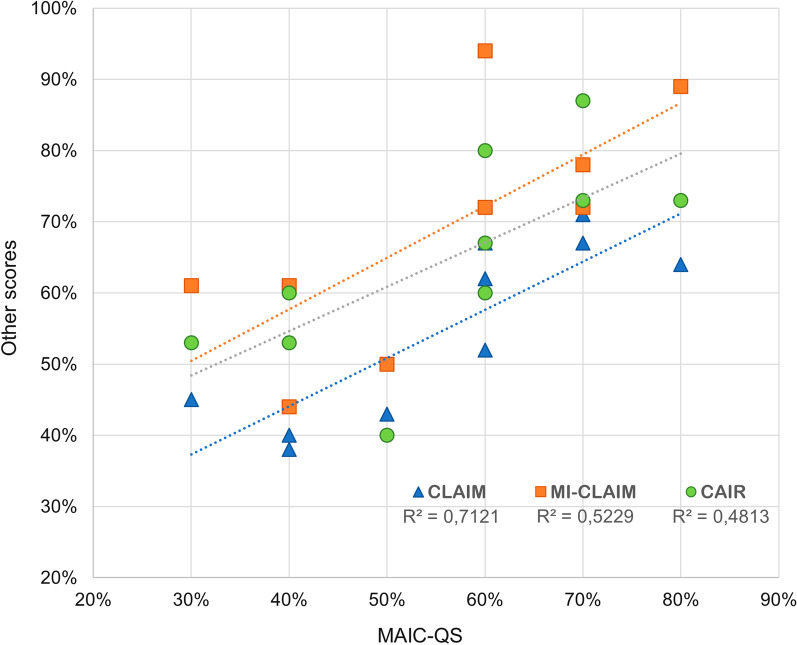
Quality scores obtained for the 10 selected articles using each of the four compared checklists. MAIC-QS is shown in the X-axis and correlations to CLAIM, MI-CLAIM, and CAIR-calculated scores are indicated. Quality scores are detailed in Additional file 1

At a final step, the 10 selected articles were also reviewed according to the selected checklists (CLAIM, MI-CLAIM, RQS, CONSORT-AI, SPIRIT-AI, and CAIR). During this process, some checklists were found to have a limited scope which made many of the items not applicable to the selected studies. CONSORT-AI and SPIRIT-AI are strictly addressed to the evaluation of studies in the form of a clinical trial and, therefore, they were considered as non-comparable to MAIC-10. On the other hand, RQS was designed to assess studies with a radiomics methodology (i.e. radiomics feature extraction and modelling) and, consequently, some items do not apply to deep learning workflows. Given these considerations, we performed a quantitative comparison of checklists CLAIM, MI-CLAIM, and CAIR against MAIC-10 by interpreting the rate of compliance based on the articles’ quality scores, expressed as percentages for comparability reasons. The results of this comparison are detailed in Fig. 1 and Additional file 1. Overall, MAIC-10 quality scores showed a good correlation with those calculated using CLAIM, MI-CLAIM, and CAIR (*R*^2^ = 0.71, 0.52, and 0.48, respectively). These scores were obtained with a reduction in checklist complexity (number of items) of 76, 47 and 33%, respectively.

Results of the intra-observer reproducibility study were obtained for each of the four checklists in terms of the repeatability coefficient, resulting in a value of RC equal to 0.22, 0.34, 0.28, and 0.12 for CLAIM, MI-CLAIM, CAIR, and MAIC-10, respectively. As smaller values of this coefficient correspond to more highly reproducible results, this finding supports our argument in favour of using the MAIC-10 checklist due to its simplicity, robustness, and intra-observer reproducibility.

## Discussion

In recent years, both medicine and medical imaging have undergone major advances, mainly due to the technological evolution of computer systems, and the development of software solutions that are ever more applicable to pattern recognition and clinical management improvements. As an example, deep learning tools can be used in different stages of a workflow to improve data quality, diagnose different illnesses, detect and segment lesions, and even predict clinical endpoints [2]. All these aspects indicate a versatile and useful instrument to work with. This progress represents a paradigm shift in the categorisation of clinical tools. Indeed, the impact of these new computational solutions on patient diagnosis, management, and prognosis is comparable to that of other devices applied in everyday life, such as interventional catheters, printed prostheses, or monitoring devices. Multidisciplinary communication and teamwork become essential to develop and apply software as medical devices (SaMDs), since clinical, imaging, and technical knowledge is needed to set an AI solution to a clinical problem [25].

Today’s omnipresence of AI tools in medicine has given rise to the concept of SaMDs, which defines any IT solutions that have an impact on the clinical aspects of the patient. In medical imaging, main areas of focus on SaMDs solutions are related to diagnosis and prognosis estimations. However, as with physical devices, digital tools require strict quality control before their approval for use in routine clinical practice. As AI software becomes more common, it is essential to establish guidelines to facilitate and ensure the correct use and usefulness of these devices in clinical practice.

Institutions such as the European Parliament, the FDA (Food and Drug Administration), and the IMDRF (International Medical Device Regulators Forum) administrations are responsible for defining, classifying, and regulating the use of SaMDs [27]. At the same time, in terms of the development and adequate publication of these solutions, different entities have developed criteria they consider essential to guarantee minimum quality standards. It is mandatory that the clinical usefulness of the output variables is prioritised in the SaMD development framework, and that the results obtained are consistent and reproducible in the general population [27]. Systematically reflecting these parameters in publications on AI tools will allow the objective analysis of the usefulness of the algorithms and their applicability in the real-world clinical scenario for which they are intended.

Our developed quality checklist, MAIC-10, aims to facilitate the process while overcoming some limitations of other published checklists in the field of AI and medical imaging. As compared to these, MAIC-10 is shorter and less complex, optimises usability, and has the potential for widespread adoption. It is also explicitly designed to provide a quantitative, objective, and reproducible quality score, with a broad scope of application across studies on AI in medical imaging, unlike SPIRIT-AI, CONSORT-AI, or RQS. Remarkably, on the set of 10 selected Insights into Imaging articles used for validation, MAIC-10 achieved a high correlation score to CLAIM, a widely used 42 items checklist. In terms of their intra-observer reproducibility, MAIC-10 was also the highest rated, being CLAIM the second highest reproducible checklist.

After reviewing the selected papers, some quality aspects seem not to be comprehensively addressed when reporting AI image-based tools. For instance, the sample size estimation is usually overlooked when developing AI algorithms due to the complexity of the task, which is evidenced in its scarcity in medical imaging publications [28]. The objective is not to discard research on small data sets, but to understand the limitations and biases of the obtained models, such as lack of generalizability and trustworthiness. A recent publication provides a practical solution for sample size estimation in the context of AI models and medical imaging [29].

On another note, anonymisation is generally not reported as a relevant part of data pre-processing, not considering data protection regulatory aspects. AI studies using clinical data should progress towards transparent reporting of data protection protocols in line with these advances, with emphasis on defining de-identification pipelines and traceability documentation.

As a limitation of our work, it must be noted that the mere publication of a quality checklist cannot be taken as a guarantee of a tangible impact in the scientific community. Instead, it must be accompanied by efforts to foster its use by both authors and reviewers, via initiatives to improve adoption and endorsement by journals [30]. Additionally, the scope of a quality assessment tool should not be overstated, as strict adherence to a checklist is not a substitute for scientific quality in a study [29]. We also recognise that the use of sub-items introduces complexity in the MAIC-10 checklist. However, as sub-items are fewer and all have to be fulfilled before an item is approved, the final checklist has been observed to be simpler when compared to CLAIM, MI-CLAIM, and CAIR. We have also shown that MAIC-10 has a higher intra-observer reproducibility compared with the other checklists and, therefore, should be preferred for its simplicity and robustness when evaluating publications on AI developments where images and clinical impact are important aspects.

In summary, the development and inclusion of AI in the management of medical imaging has become a reality in current publications and clinical practice. The use of these new technologies requires implementing quality criteria that allow us to assess the usefulness of these approaches when they are published. Our proposed MAIC-10 unifies and simplifies the most relevant and reiterated criteria among those proposed to date, establishing a basic scheme that both authors and readers can use to help interpret publications on AI in medical imaging.

## Supplementary Information

**Additional file 1**. Scoring of each of the 10 selected articles according to checklists MAIC-10, CLAIM, MI-CLAIM, and CAIR. Two sets of scores are provided, which correspond to the results used in the intra-observer reproducibility study.

## Data Availability

All data generated and analysed in this study are included in the manuscript and additional files.

## Abbreviations

AI: Artificial intelligence
CAIR: Clinical AI research
CDM-eCRF: Common data model for electronic case report form
CLAIM: Checklist for artificial intelligence in medical imaging
CNN: Convolutional neural network
CONSORT-AI: Consolidated standards of reporting trials-AI
DECIDE-AI: Developmental and exploratory clinical investigations of decision support systems driven by artificial intelligence
DL: Deep learning
EQUATOR: Enhancing the quality and transparency of health research
FDA: Food and drug administration
IMDRF: International medical device regulators forum
MAIC-10: Must AI criteria-10
MAIC-QS: MAIC quality score
MI-CLAIM: Minimum information about clinical artificial intelligence modelling
ML: Machine learning
P-A-CS: Picture archiving and communication systems
PROBAST-AI: Prediction model risk of bias assessment tool, artificial intelligence
RQS: Radiomics quality score
SaMD: Software as a medical device
SPIRIT-AI: Standard protocol items recommendations for interventional trials
STARD-AI: Standards for reporting of diagnostic accuracy study, artificial intelligence
TRIPOD-AI: Transparent reporting of a multivariable prediction model of individual prognosis or diagnosis, artificial intelligence
